# The Impact of COVID-19 on the Job Satisfaction of Cardiothoracic Trainees in the United Kingdom: An Insight From the General Medical Council National Training Survey

**DOI:** 10.7759/cureus.73682

**Published:** 2024-11-14

**Authors:** James R O'Hanlon, Jeremy Chan, Daniel P Fudulu, Gianni D Angelini

**Affiliations:** 1 Department of Medical Education, Charing Cross Hospital, London, GBR; 2 Department of Cardiothoracic Surgery, Bristol Heart Institute, University of Bristol, Bristol, GBR

**Keywords:** cardiothoracic surgical education, covid-19, general medical council, job satisfaction, medical training, national training survey

## Abstract

Introduction

The National Training Survey (NTS) is conducted annually by the General Medical Council (GMC) to monitor and report on the quality of postgraduate medical education and training. The coronavirus disease 2019 (COVID-19) pandemic had a significant impact on training in cardiothoracic surgery due to the unprecedented restructuring of surgical activity across the UK. We aim to evaluate how the job satisfaction of cardiothoracic surgery trainees was impacted by COVID-19 using the National Training Survey.

Methods

The National Training Survey in cardiothoracic surgery from 2017 to 2024 was obtained from the General Medical Council website. Job satisfaction for trainees was evaluated using 17 indicators: Adequate Experience, Clinical Supervision, Clinical Supervision Out of Hours, Educational Governance, Educational Supervision, Feedback, Handover, Induction, Local Teaching, Overall Satisfaction, Regional Teaching, Reporting Systems, Rota Design, Study Leave, Supportive Environment, Teamwork and Workload. The pre-pandemic score for each indicator was an average of 2017, 2018 and 2019, while the post-pandemic score was an average of 2022, 2023 and 2024. The overall job satisfaction per year, combining all indicators, was also analysed.

Results

Thirty-eight cardiothoracic centres were included in this study. There was a reduction in trainees’ job satisfaction from pre- to post-pandemic surveys when comparing all 17 indicators together (scored out of 100) (73.87 versus 70.97, p<0.001). There were six out of 17 (35.29%) indicators that demonstrated a significant decrease in job satisfaction amongst trainees: Regional Teaching (70.53 versus 54.53, p<0.001), Adequate Experience (78.65 versus 72.34, p=0.003), Local Teaching (68.26 versus 62.92, p=0.037), Educational Governance (73.78 versus 70.36, p=0.033), Clinical Supervision Out of Hours (91.16 versus 87.95, p=0.005) and Clinical Supervision (91.98 versus 88.92, p=0.014).

Conclusion

Trainees’ job satisfaction significantly decreased after the COVID-19 pandemic. Although the most recent survey suggests job satisfaction amongst trainees may be recovering, further work needs to be done to ensure training standards return to pre-pandemic levels.

## Introduction

The World Health Organization (WHO) declared the coronavirus disease 2019 (COVID-19) a pandemic in March 2020 [1]. In response to this, the Royal College of Surgeons (RCS) published its guidance in March 2020 for patient selection and surgical practice across the UK [2]. The initial guidance involved the cancellation of elective operating cases, with an emphasis on emergency cases [3]. The Society for Cardiothoracic Surgery (SCTS) in Great Britain and Ireland introduced its specialised national guidelines on the performance of cardiac surgery [4]. It stratified patient selection for surgery and aimed to smooth the gradual reintroduction of elective surgery. Furthermore, cardiac services were streamlined by creating centralised units and combining the patient population into fewer cardiac units, such as the North‐West Urgent Cardiothoracic Service (NUCS) and Pan London Emergency Cardiac Surgery (PLECS) [4,5]. As a result, there was a significant reduction in operational activities, by as much as 83% in some cardiac surgical units [6]. The effect was also seen globally, with a 50%-75% reduction in cardiac activity reported during the pandemic across 60 centres in a worldwide-based survey [7]. Inevitably, cardiothoracic trainees would have less surgical experience during the height of the COVID-19 pandemic in the UK.

The General Medical Council (GMC) has conducted the National Training Survey (NTS) annually since 2006 to monitor and report on the quality of postgraduate medical education and training in the UK [8]. Further information regarding speciality and core training applications has been made available since 2016. More than 74,000 doctors, trainers and trainees in GMC-approved training posts completed the NTS in 2024, making this one of the largest postgraduate training surveys [9]. In this paper, we aim to evaluate how the job satisfaction of cardiothoracic surgery trainees was impacted by COVID-19 using the GMC-NTS.

## Materials and methods

The NTS report on cardiothoracic surgery from 2017 to 2024 was obtained from the online reporting tool on the GMC website [8]. The reports are open access, and a summary of the latest 2024 questionnaire is publicly available [10]. For each year, the surveys were collected between March and April, and then, the results were published in July for that respective year. The data from the NTS from 2017 to 2024 was extracted and analysed in September 2024. The method was previously described by Chan et al. [11]. The NTS contained several questions that were grouped into different indicators, each of which was scored out of 100. There were 19 indicators identified between 2017 and 2024; however, two were excluded (Curriculum Coverage and Facilities) as neither featured both pre- and post-pandemic surveys to create a comparison. This left 17 indicators that evaluated job satisfaction amongst cardiothoracic trainees (Table 1). The results were collected as an aggregate value of the indicator scores from each trust.

**Table 1:** Seventeen indicators in the General Medical Council National Training Survey for trainees

Sr. No.	Indicators in the General Medical Council National Training Survey
1.	Adequate Experience
2.	Clinical Supervision
3.	Clinical Supervision Out of Hours
4.	Educational Governance
5.	Educational Supervision
6.	Feedback
7.	Handover
8.	Induction
9.	Local Teaching
10.	Overall Satisfaction
11.	Regional Teaching
12.	Reporting Systems
13.	Rota Design
14.	Study Leave
15.	Supportive Environment
16.	Teamwork
17.	Workload

The online reporting tool did not give access to the total number of trainees. Instead, for each individual score from a trust or deanery, they reported a respondent range. Therefore, the total number of trainees for each year was calculated as a total of the respondent ranges reported in the online reporting tool.

Comparisons in all 17 indicators for trainees were made before the declaration of the COVID-19 pandemic in March 2020 and after the restrictions were lifted in July 2021. The pre-pandemic mean scores were an average of 2017, 2018 and 2019, while the post-pandemic mean scores were an average of 2022, 2023 and 2024. The NTS in 2020 and 2021 were excluded as they took place during the pandemic restrictions. National mean scores were an average of all six years collected. The scores from all indicators were compared on a year-by-year basis.

**Figure 1:**
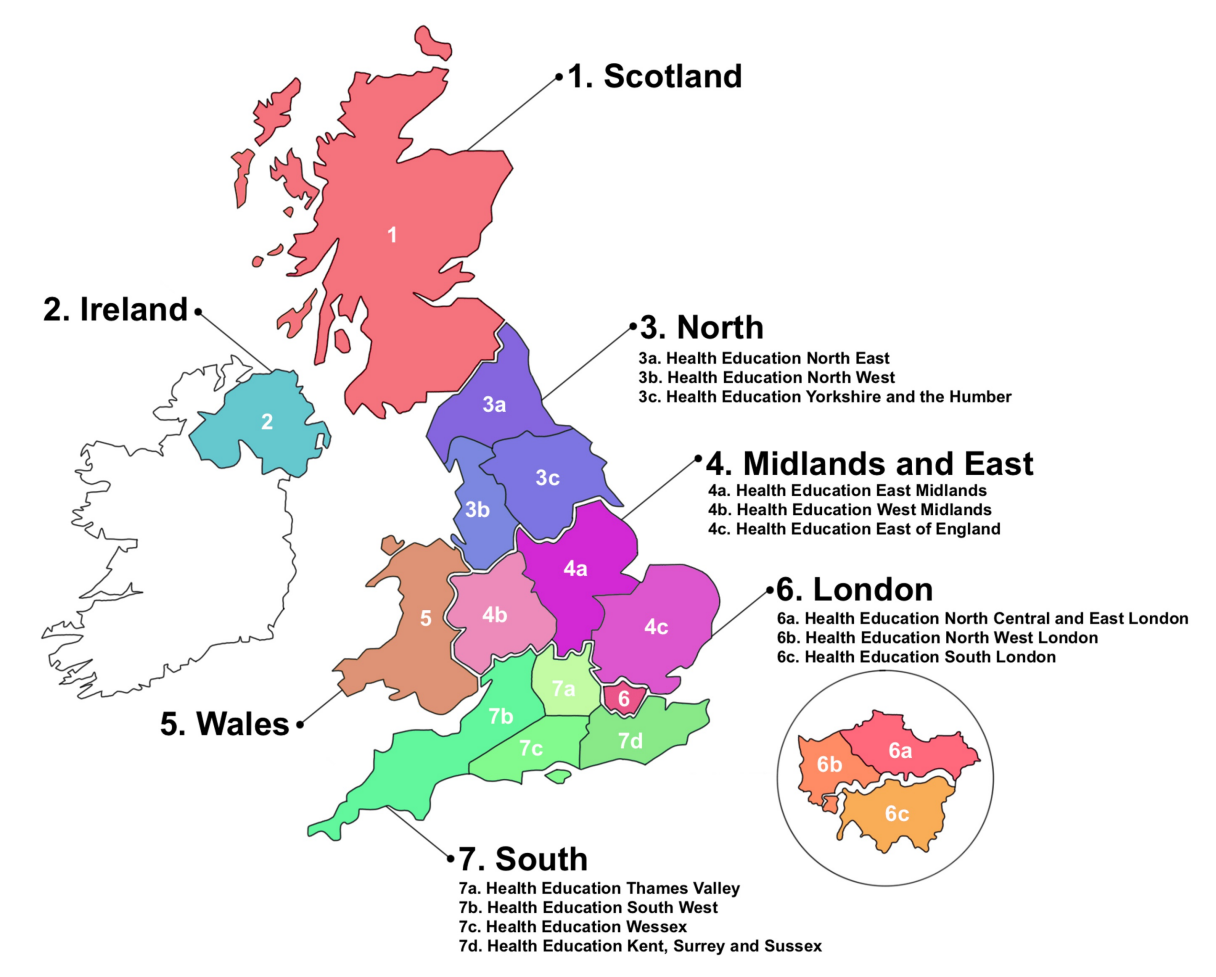
Seven regions identified in our study Centres in England were divided into four regions based on the LETBs. The deaneries within these are identified. LETBs: local education training boards

The cardiothoracic centres were then further analysed according to geographical location. The centres were divided into England, Scotland, Wales and Northern Ireland. Centres in England were further divided into four regions based on the local education training boards (LETBs): London, Midlands and East, North and South. The seven regions analysed, including the deaneries within it, are summarised in Figure 1.

Analysis

IBM® SPSS® Statistics for Windows version 25 (IBM Corp., Armonk, NY) and Microsoft Excel 365 (Microsoft Corp., Redmond, WA) were used for statistical analysis. The scores of each of the trusts in each year were extracted from the online reporting tool on the GMC website and used for further statistical analysis. The Mann-Whitney U test was calculated in SPSS and used to determine the significance between the aggregate indicator scores from pre-pandemic and post-pandemic data. A p‐value of less than 0.05 was deemed to be statistically significant.

## Results

Thirty-eight cardiothoracic centres were included in this study, of which 32 are in England, three in Scotland, two in Wales and one in Northern Ireland. The total range of trainees included in the GMC-NTS for each year is shown in Table 2.

**Table 2:** Total range of cardiothoracic trainees in each year

Year	Range of cardiothoracic trainees
2017	102-150
2018	99-145
2019	91-135
2022	55-85
2023	65-105
2024	55-85

The top three indicators that trainees were most satisfied with across the six years were Clinical Supervision (90.66), followed by Clinical Supervision Out of Hours (89.79) and finally Educational Supervision (86.74). The least satisfied areas were Workload (45.77), Rota Design (60.92) and Regional Teaching (63.86). The national means for all 17 indicators are shown in Table 3.

**Table 3:** Pre-pandemic, post-pandemic and national six-year means of all indicators for trainees

Indicator	Six-year mean	Pre-pandemic mean	Post-pandemic mean	Difference	p-value
Adequate Experience	75.90	78.65	72.34	-6.31	0.003
Clinical Supervision	90.66	91.98	88.92	-3.07	0.014
Clinical Supervision Out of Hours	89.79	91.16	87.95	-3.21	0.005
Educational Governance	72.29	73.78	70.36	-3.42	0.033
Educational Supervision	86.74	86.79	86.67	-0.12	0.793
Feedback	77.27	78.49	75.82	-2.67	0.210
Handover	64.05	64.48	63.47	-1.02	0.627
Induction	77.18	77.75	76.44	-1.31	0.535
Local Teaching	66.14	68.26	62.92	-5.35	0.037
Overall Satisfaction	76.25	77.93	74.08	-3.85	0.059
Regional Teaching	63.86	70.53	54.53	-16.00	<0.001
Reporting Systems	72.65	74.09	70.77	-3.32	0.095
Rota Design	60.92	62.78	59.33	-3.45	0.199
Study Leave	64.34	63.42	65.54	2.12	0.559
Supportive Environment	69.21	68.52	70.09	1.56	0.318
Teamwork	75.77	75.50	76.10	0.60	0.527
Workload	45.77	46.44	44.90	-1.54	0.445
Total	72.61	73.87	70.97	-2.91	<0.001

**Figure 2:**
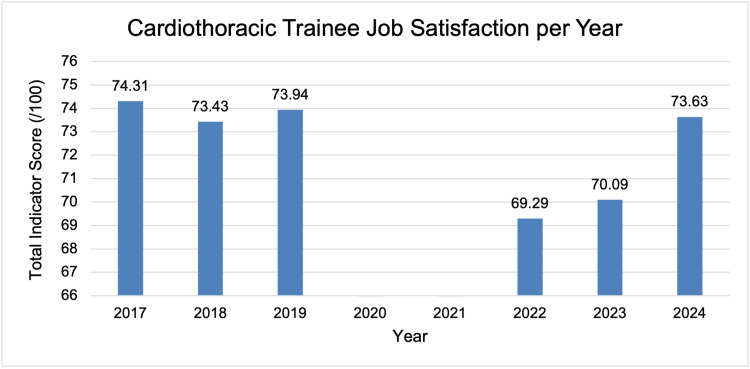
Overall satisfaction of cardiothoracic trainees from 2017 to 2024

There was a significant reduction in trainees’ overall job satisfaction comparing pre-pandemic and post-pandemic surveys for all indicators evaluated (73.87 versus 70.97, p<0.001) (Table 3). The largest difference in job satisfaction occurred after the pandemic, between 2019 and 2022, with a score of 73.94 and 69.29, respectively (Figure 2). There is then a steady increase in trainees’ job satisfaction towards pre-pandemic levels in the years following.

Fourteen indicators demonstrated a decreasing trend in job satisfaction amongst trainees from pre-pandemic to post-pandemic years, six of which were statistically significant (Table 3). The three areas with the largest decrease in job satisfaction were Regional Teaching (70.53 versus 54.53, p<0.001), Adequate Experience (78.65 versus 72.34, p=0.003) and Local Teaching (68.26 versus 62.92, p=0.037), all of which were significant. The other indicators that demonstrated a significant decrease were Educational Governance (73.78 versus 70.36, p=0.033), Clinical Supervision Out of Hours (91.16 versus 87.95, p=0.005) and Clinical Supervision (91.98 versus 88.92, p=0.014).

The regions with the highest job satisfaction amongst trainees across the six years were London (77.53), South (77.13) and Midlands and East (76.65) (Table 4). The regions with the lowest job satisfaction were Wales (73.30), Ireland (73.55) and North (73.60). All seven regions saw a decrease in their job satisfaction levels amongst trainees since the pandemic, of which Wales (77.33 versus 70.04, p=0.014), Ireland (76.54 versus 69.25, p=0.012) and Midlands and East (80.11 versus 73.52, p<0.001) were significant. In the post-pandemic years analysed, there was a significant difference between London and Ireland (p=0.023), London and Wales (p=0.010), South and Ireland (p=0.031) and South and Wales (p=0.032). Otherwise, there was no statistical significance between the regions after the pandemic.

**Table 4:** Pre-pandemic, post-pandemic and six-year means of all seven regions

Region	Six-year mean	Pre-pandemic mean	Post-pandemic mean	Difference	p-value
London	77.53	78.91	76.42	-2.49	0.224
South	77.13	78.43	75.55	-2.88	0.336
Midlands and East	76.65	80.11	73.52	-6.58	<0.001
Scotland	73.91	74.34	73.58	-0.75	0.869
North	73.60	73.74	73.50	-0.24	0.428
Ireland	73.55	76.54	69.25	-7.28	0.012
Wales	73.30	77.33	70.04	-7.28	0.014

## Discussion

Our result shows that cardiothoracic surgical trainees’ overall job satisfaction has reduced significantly after the COVID-19 pandemic. There is a significant reduction in six out of 17 (35.29%) individual quality indicators reported in the National Training Survey.

Job satisfaction has been defined as the positive emotional state one acquires from their work [12]. Numerous factors affect job satisfaction for surgical trainees, including autonomy, training opportunities, excessive workload, adequate supervision and collegiality [13-15]. Importantly, satisfied doctors demonstrate better performance [16]. The NTS demonstrated that job satisfaction amongst trainees significantly decreased after the pandemic. The year with the least job satisfaction was the first post-pandemic year (Figure 2). Since then, there has been a steady recovery of job satisfaction back to pre-pandemic levels. We have identified four key themes that can be addressed to help recover job satisfaction amongst trainees: (1) Training Experience, (2) Local and Regional Teaching, (3) Educational Governance and (4) Supervision.

Surgical trainees reported a significant decrease in their satisfaction with adequate experience in their training, according to the NTS. The restructuring of surgical services across the NHS, due to COVID-19, had a profound impact on surgical experience in the UK [17]. In London, the Society of Cardiothoracic Surgeons PLECS group advised that during COVID-19, only two of the seven cardiac surgical centres should have a cardiac surgical service [4]. At St. Bartholomew’s Hospital, one of the London centres that remained open, Abdel Shafi et al. reported that training opportunities were greatly diminished [18]. They attributed this to a 59.3% overall reduction in the number of cardiac operations performed. This was also combined with an increased proportion of the remaining high-risk patients being operated on by consultants, due to complications from COVID-19. The key outcome of their study was the number of trainee-led operations in cardiac surgery significantly decreased during the COVID-19 pandemic. Similar restructuring of cardiac services was seen across the country, such as the NUCS [4]. Clements et al. analysed the quantitative impact of COVID-19 on surgical training in the UK [19]. They demonstrated that a significant reduction in recorded operative experience was observed in 2020 compared with 2019 for all surgical trainees. In cardiothoracic surgery, only 64% of surgeries were being logged in 2020 compared to 2019 in the same timeframe. Additionally, they found that trainees struggled to meet their competency requirements during the pandemic. Over one in eight final-year surgical trainees had to have their training extended as they were unable to gain their competencies due to the pandemic.

The NTS revealed that trainees became significantly dissatisfied with both local and regional teaching in the UK after the pandemic. Caruana et al. found that the majority of cardiothoracic trainees (57%), in a UK national survey, felt that their current role offered little to no relevant learning opportunities and was a deterrent to their professional development during COVID-19 [20]. There was also very poor documentation of learning encounters during COVID-19, with only very few trainees having completed a workplace assessment and the majority commenting that it was unachievable.

The third theme identified in the analysis was that trainees have become more dissatisfied with educational governance, especially in raising concerns about their education and training. The NTS assessed this by asking trainees whether they felt confident in raising concerns about their education and training, and if they felt it would be addressed. The importance of raising concerns in medicine, and understanding the challenges in doing so, is well researched [21]. Specific concerns highlighted in the literature for surgical training include bullying and harassment [22]. Sexual misconduct is another troubling issue within surgery, with women disproportionately affected [23]. The consequence of this leads to a poor learning environment for trainees [22]. The most common barriers to raising a concern include the following: nothing will be done, fear that it will cause a negative effect on working relationships and your career or that a complaint will be made about yourself [24]. Denning et al. demonstrated that COVID-19 contributed to an underreporting of incidences possibly due to an increased workload and a change in the perceived relative importance of incidences [25].

Finally, our report has also highlighted that trainees have become significantly dissatisfied with supervision, both in and out of hours. Adequate supervision throughout training has been demonstrated to improve not only patient outcomes but also trainee satisfaction and learning [26]. Numerous studies have demonstrated a disruption to supervision due to the pandemic [27-29]. In an international study of 86 plastic surgery residents, only 39% of the trainees reported receiving adequate supervision during the pandemic [27]. Healthcare workers expressed their need for more support during the COVID-19 pandemic, which remained largely unmet by supervisors [28]. However, it was also found that supervisors themselves experienced a great deal of stress during the pandemic and needed support to meet their own needs. It has been recommended that regular communication from current supervisors, as well as a supervisor in the new department, should be implemented if any redeployment is needed for future NHS crises [29].

All regions across the UK saw a decrease in job satisfaction amongst trainees, most significantly in Wales, Ireland and Midlands and East. The research of Clements et al. on the quantitative impact of COVID-19 on surgical training also addressed regional variance across the UK [19]. Similarly, they found that all regions of the UK were severely affected by the number of operations performed. In particular, they found Wales and West Midlands amongst the most affected regions. An uneven distribution in the reduction of surgical operations across the UK could be one of many contributing factors to the regional variation in job dissatisfaction.

Fitzgerald et al. in 2011 discussed the regional variations in surgical education across the UK using the Membership of the Royal Colleges of Surgeons of Great Britain and Ireland (MRCS) Objective Structured Clinical Examination (OSCE) examination results [30]. They discovered that the examination pass rate varied across the nation, ranging from 54% to 94%, suggesting a regional variation in trainee performance. Rodrigues et al. also identified a regional variation in surgical exposure in national ophthalmology training [31]. This raises the importance of recognising regional variation in surgical training to ensure trainees are not unfairly disadvantaged purely due to geographical location. Our study supports the idea that there is regional variation in job satisfaction amongst trainees in the UK.

Limitations

Staff, associate specialists and specialty doctors (SAS) doctors, which represent a significant proportion of the workforce in cardiothoracic surgery, were not included in the GMC National Training Survey. According to the GMC, one in six doctors are working as an SAS doctor in the UK. Moreover, SAS doctors tend to work more regular hours than cardiothoracic trainees to maintain services [32]. Further work should consider integrating both trainees and SAS doctors’ opinions.

Compared with other surgical specialties, the number of cardiothoracic centres and trainees in cardiothoracic surgery remains low. Cardiothoracic centres that did not have survey responses from more than three trainees were not included in the overall averages in the GMC-NTS to maintain anonymity. Therefore, cardiothoracic surgeons’ responses may have been excluded from the analysis. Additionally, we were unable to view the exact number of trainees in the study, as the online reporting tool only reports respondent ranges.

The COVID-19 pandemic was undoubtedly the most significant disruption to medical training in 2020 and 2021, and therefore one of the most likely reasons for reduced job satisfaction. However, other confounding factors during this period may have contributed to the disruption of cardiothoracic training. This could include longstanding issues such as the limited number of consultants available for training, the reduction in working hours and surgical operative experience, or more recent issues such as the 2023 junior doctor strikes [33,34].

## Conclusions

Job satisfaction amongst cardiothoracic trainees decreased significantly after the pandemic but has shown some signs of returning to pre-pandemic levels. Our analysis indicates the underlying causes were in Training Experience, Local and Regional Teaching, Educational Governance and Supervision. These factors that influence job satisfaction for cardiothoracic trainees are also shared amongst many other professions. Therefore, strategies used to improve job satisfaction outside of medicine could be utilised within the NHS.

Cardiothoracic training centres should reflect on their trainees’ evaluations and look to improve on specific areas if deemed unsatisfactory. This will not only aim to improve morale amongst trainees but also patient outcomes. Further work needs to be done by addressing deficiencies in training, such as the ones highlighted in this paper, and implementing opportunities for trainees to rectify the damage caused by COVID-19 to ensure training standards return to pre-pandemic levels.
